# Fixed-Bearing Unicompartmental Knee Arthroplasty of the Lateral Compartment: A Series of 246 Cases

**DOI:** 10.1016/j.artd.2023.101183

**Published:** 2023-09-09

**Authors:** Michael Fitzsimons, Johan van der Stok, Joseph M. Queally, Turlough O'Donnell

**Affiliations:** a The Centre for Orthopaedics, Beacon Hospital, Dublin, Ireland; b UCD School of Medicine, University College Dublin, Dublin, Ireland

**Keywords:** Arthroplasty, Fixed-bearing implant, Gonarthrosis, Unicompartmental knee arthroplasty

## Abstract

**Background:**

Isolated osteoarthritis of the lateral compartment of the knee is less common than that of the medial compartment, resulting in significantly fewer lateral unicompartmental knee arthroplasties (UKAs) being performed. This study aimed to evaluate results of a fixed-bearing UKA for the treatment of lateral compartment osteoarthritis of the knee.

**Methods:**

A prospectively collected cohort of 255 patients undergoing fixed-bearing UKA of the lateral compartment using the Triathlon PKR (Stryker, Warsaw, IND) implant with a minimum 2-year follow-up was reviewed. The Western Ontario and McMaster Universities Osteoarthritis Index (WOMAC) score, radiographic alignment, complications, reoperations, and revisions were recorded. Patient factors and pre- and post-surgical alignment were assessed for their association with a minimum important change (MIC) of the total WOMAC score.

**Results:**

A total of 246 implants with a mean follow-up of 6.6 years (2-10.8 years) were included (4% lost to follow-up). The total WOMAC score increased from 61.3 ± 3.5 to 85.3 ± 7.5, exceeding the MIC in 215 patients (88%). Exceeding the MIC was not associated with age, body mass index, or alignment. The 5-year implant revision rate was 1.6% (3/187).

**Conclusions:**

The fixed-bearing Stryker Triathlon PKR implant for lateral UKA resulted in good clinical outcomes with a low revision rate at midterm follow-up. Body mass index, age, and pre- and post-surgical alignment did not correlate with the clinical outcome. Long-term follow-up is necessary to determine if the clinical improvement and low revision rate can be maintained.

## Introduction

Unicompartmental osteoarthritis (OA) of the lateral compartment is seen in between 8% and 15% of patients with radiographic changes of OA [1,2], and <10% of unicompartmental knee arthroplasties (UKAs) are performed for disease of the lateral compartment, encompassing 1% of all knee arthroplasties [3]. Lateral UKA is considered technically more demanding than medial UKA, both from a surgical as well as an implant perspective [4]. The lateral compartment differs anatomically, and this result in a far greater backward sliding of lateral femoral condyle during flexion compared to the medial side [5]. There is also an increased risk of patellar impingement due to lateral tracking of the patella in flexion [6]. The lateral condyle is also internally rotated with respect to the axial plane, contributing to the “screw home” mechanism in extension of the knee [6].

The implant types used for lateral UKA cope with these kinematic changes in different ways [7]. The original Oxford design of the medial mobile-bearing UKA was used in lateral UKA [8]. Despite success in medial UKA, initially high rates of bearing dislocation were reported, and this was attributed to the posterior femoral translation on the lateral condyle and the laxity of the lateral collateral ligaments in flexion [7]. Although the lateral mobile-bearing UKA design has since been improved to reduce this risk of bearing dislocation [7], others have focused on the design of fixed-bearing implants, which negate this risk of bearing dislocation. However, due to a relative lack of congruence with a fixed-bearing implant, which does not replicate the native knee kinematics like mobile-bearing designs, concerns existed regarding polythene wear [9]. Nowadays, this risk has been shown to have become significantly decreased [10], likely due to improved wear properties of highly cross-linked polyethylene [11]. This makes fixed-bearing implants a viable option for the patients with isolated OA of the lateral compartment.

Historically, lateral UKA was associated with poorer outcomes and higher revision rates compared to medial UKA [4]. But results from more recent studies and joint registry data demonstrate that there is now little difference in terms of clinical outcome and survivorship [[12], [13], [14]]. However, the strength of these findings remains limited by the restricted quality and quantity of studies available that provide mid- to long-term data on lateral UKA [14]. Furthermore, there are only a small number of series reporting outcomes for fixed-bearing implants in lateral compartment disease [15], despite recent evidence reporting superior survivorship in comparison to mobile-bearing implants [13,16]. In this study, we report midterm clinical outcome and revision rates of a large cohort of lateral UKAs using a fixed-bearing implant (Triathlon PKR implant, Stryker). Our hypothesis was that fixed-bearing UKA implants for lateral OA can lead to a significant improvement in patient-reported outcome with a low revision rate. We also aimed to identify patient- or surgery-related factors, including age, body mass index (BMI), and alignment, that correlated with a clinically relevant improvement in the functional outcome.

## Material and methods

Approval from the institutional review board was obtained for this study. A prospectively collected hospital database including all lateral UKA procedures performed between September 2010 and July 2018 was reviewed. Indications for a lateral UKA were: OA of the lateral compartment; asymptomatic OA of patellofemoral compartment; correctable deformity in the coronal plane; and stability. Contraindications were: OA of the medial compartment; symptomatic OA of patellofemoral compartment (defined as anterior knee pain on flexion or direct compression of patellofemoral joint); fixed flexion deformity >5°; and inflammatory arthritis. All surgeries were carried out by a single surgeon, fellowship-trained in the technique (T.O.D.).

### Surgical technique and rehabilitation

**Figure 1:**
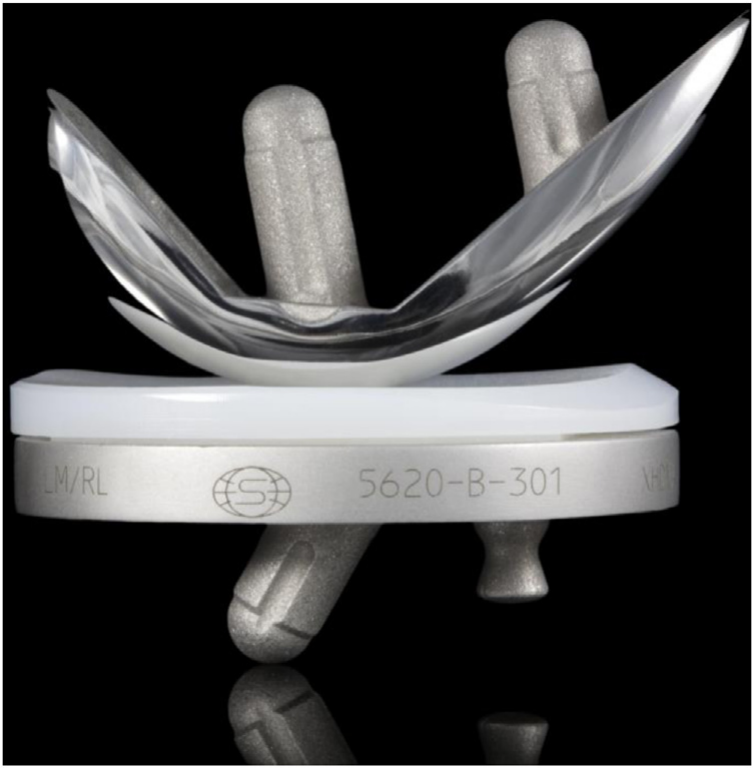
Image of the Triathlon PKR implant (Stryker, Warsaw, IND).

Surgery was performed using the Triathlon PKR implant (Stryker, Warsaw, IND) (Fig. 1). The Triathlon PKR is a cemented implant with a metal-backed fixed-bearing made of highly cross-linked polyethylene.

The patient was positioned supine with a lateral thigh support and a footrest. A tourniquet was used in all cases. The skin incision was made lateral to the midline, followed by a lateral parapatellar arthrotomy. Then, minimal resection of the anterior fat pad and limited release of the lateral collateral ligament on the tibia are performed. Care should be taken with retractors, especially laterally, as injury to the common peroneal nerve is reported [8]. The tibial cut is planned with extramedullary alignment, and appropriate resection depth and recreation of the patient’s natural posterior tibial slope are set prior to cutting. Transverse cuts were made using the cutting guide. Special care was taken to acquire the correct alignment of the sagittal cut (20-25° of internal rotation with respect to the midline), which requires retraction of the patellar tendon. Osteophytes were removed at this point prior to soft tissue balancing. Flexion and extension gap balancing was performed, aiming to correct to neutral or minimal valgus (<2°) alignment. The distal femoral cut was performed with the distal resection guide, followed by the posterior condyle and chamfer resections. Satisfactory balance was assessed with the trial implants and spacers. Final peg preparation of the femur and tibia was performed, and the definitive components were subsequently implanted using a single cementing technique (Palacos, Heraeus, Wehrheim, Germany), with particular care taken to remove cement remnants laterally and posteriorly.

After implantation of the insert, the knee was brought to 30° of flexion, and a valgus stress was applied to allow for pressurization during maturation of the cement. A drain was left in situ for 24 hours. A minimal facetectomy of the patella was performed, although not in all cases. The retinaculum was closed with the aid of a suture anchor in the lateral facet of the patella, and the subcutis and skin were closed in layers and a pressure bandage was applied.

All patients were rehabilitated using a hinged knee brace allowing 70° of flexion and full weight-bearing for the first 2 weeks postoperatively. After 2 weeks, the brace was removed and unrestricted full weight-bearing was allowed. Patients were encouraged to start cycling and hydrotherapy at that point. Running was not encouraged but advised after 3 months if patients so wished, and returning to unlimited activity after 6 months.

### Data collection

The following patient- and surgery-related data were recorded prior to surgery: age, sex, BMI, affected side, duration of symptoms, previous surgery, and alignment (measured as the hip-knee-ankle [HKA] axis on a standing long-leg radiograph). The functional outcome was measured using the Western Ontario and McMaster Universities Osteoarthritis Index (WOMAC) score with a 5-point Likert scale. WOMAC scores were collected presurgery and at 6-week, 6-month, and final follow-up. Furthermore, complications, reoperations, and revisions were recorded.

### Statistical analysis

Statistical analysis was carried out using GraphPad Prism 9.1.1 (San Diego, CA, USA). Continuous variables were stated as mean +standard deviation and median (minimum-maximum); categorical variables were stated as number (*n*) and percentage (%). To analyze differences in WOMAC scores over time, a Friedman test with a Dunn's correction for multiple comparisons (time points) was used, thereby excluding patients that were lost to follow-up. To determine patient factors associated with a clinical outcome, a multivariate logistic regression model was applied. Clinical outcome was categorized as ‘clinically relevant improved‘ or ‘no clinically relevant improved’, using the minimum important change (MIC) values for the WOMAC score as described by Clement *et al*: 21 for pain, 13 for stiffness, 16 for function, and 17 for the total WOMAC score [17]. WOMAC scores were standardized as a percentage in a reverse format consisting of a range from 0 (worst) to 100 (best) in accordance with Singh *et al* [18]. The data were presented as odds ratio with a 95% confidence interval. A *P*-value of <.05 was considered statistically significant.

## Results

In total, 255 patients underwent a lateral UKA between September 2010 and July 2018. Patient demographics are shown in Table 1. The mean follow-up was 6.6 years (2-10.8 years), and 9 patients (4%) were lost to follow-up.

**Table 1:** Patient characteristics.

	*n* = 255
Age (y)	57 ± 8 (range 27-79)
BMI (kg/m^2^)	28.5 ± 3.1
Sex	
Male	125 (49%)
Female	130 (51%)
Duration of symptoms (mo)	21 ± 10
Side	
Right	130 (51%)
Left	125 (49%)
Prior surgery	
Arthroscopy	84 (33%)
Anterior Cruciate Ligament (ACL) reconstruction	1 (0.3%)
Lateral Collateral Ligament (LCL) repair	1 (0.3%)
Follow-up (y)	6.6 ± 2.2

### Pre- and post-surgery alignment

**Figure 2:**
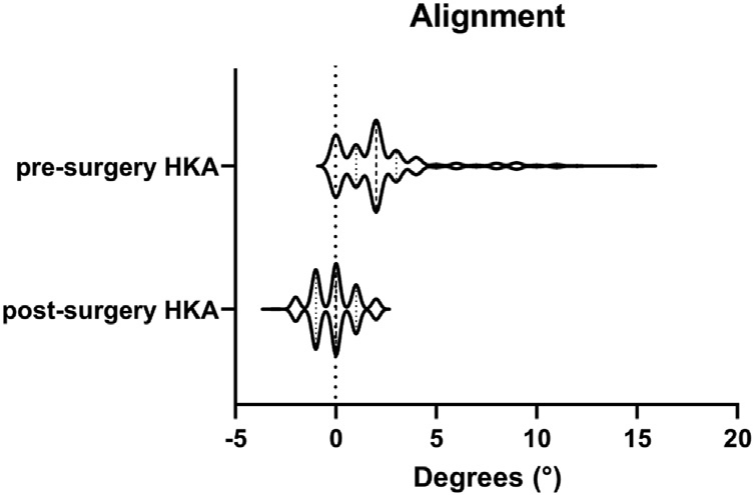
Box-plot of pre- and post-operative alignment.

**Figure 3:**
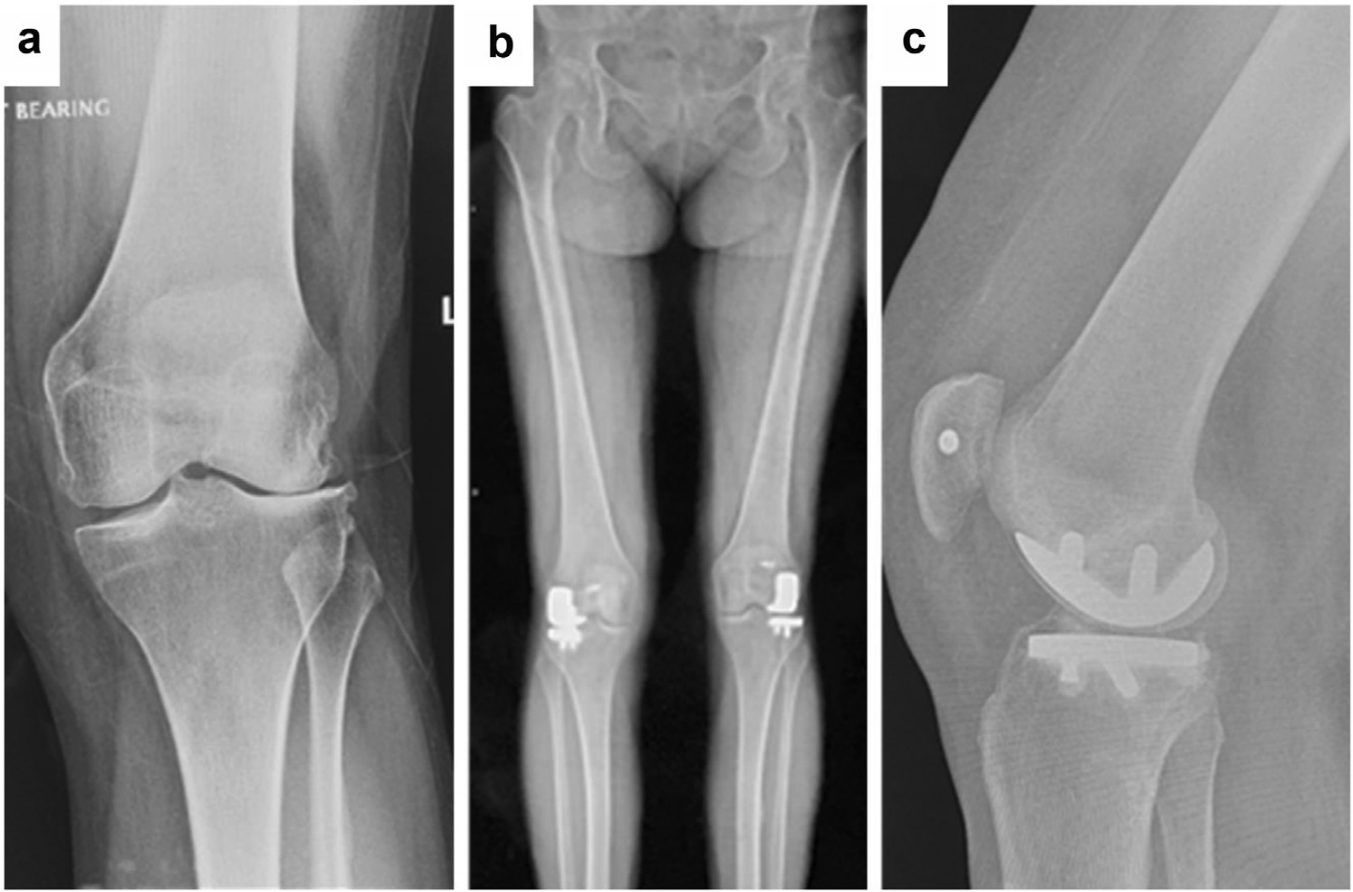
Pre- and post-operative radiograph of patient with isolated lateral unicompartmental OA who underwent a lateral UKA using the Triathlon PKR implant. (a) Preoperative anterior-posterior radiograph, (b) postoperative anterior-posterior radiograph, (c) postoperative lateral radiograph.

The presurgical alignment, measured using the HKA-axis, ranged from neutral to 15° of valgus (Fig. 2). Most knees showed a mild valgus (1°-2°, 49%) presurgery (Table 2). The average correction achieved during surgery was −2.5° ± 2.8°, with a maximum correction of 15° (Fig. 3) The postsurgery alignment was a mild varus (−2° to −1°, 39%), neutral (0°, 35%), or a mild valgus (1°-2°, 26%).

**Table 2:** Alignment.

	HKA-axis	Presurgery	Postsurgery
Mild varus	−2° to −1°	0	100 (39%)
Neutral	0°	58 (23%)	88 (35%)
Mild valgus	1°-2°	124 (48%)	67 (26%)
Moderate valgus	3°-4°	45 (18%)	0
Severe valgus	>5°	28 (11%)	0

### Clinical outcome

The WOMAC score of patients showed a significant improvement during follow-up (Table 3). Final scores of the pain, flexion, and function subdomains were 16.6 ± 2.5, 8.0 ± 0.9, and 60.8 ± 4.6, respectively, and the total WOMAC score increased from 61.3 ± 3.5 to 85.3 ± 7.5, an increase of 39% (*P* < .001). The subdomain scores for pain, flexion, and function showed clinically relevant improvements in 227 (93%), 136 (56%), and 162 (66%) patients, respectively, and 215 patients (88%) showed a clinically relevant improvements in the total WOMAC score. A multivariate logistic regression model showed no correlation between the patients that exceeded the MIC for the total WOMAC score and age, sex, BMI, or pre- and post-surgical alignment (Table 4).

**Table 3:** WOMAC score.

	Presurgery (*n* = 255)	6 wk (*n* = 255)	6 mo (*n* = 255)	Final (*n* = 242)	*P*-value
WOMAC - pain	6.1 ± 1.2	11.1 ± 2.3	13.9 ± 2.8	16.6 ± 2.5	<.001
WOMAC - flexion	6.2 ± 0.9	7.0 ± 1.2	6.9 ± 0.8	8.0 ± 0.9	<.001
WOMAC - function	48.9 ± 2.6	49.6 ± 5.8	54.3 ± 4.2	60.8 ± 4.6	<.001
WOMAC - total	61.3 ± 3.5	67.6 ± 8.1	75.1 ± 6.8	85.3 ± 7.5	<.001

Friedmann test with Dunn’s correction.

**Table 4:** Multiple logistic regression analysis of exceeding MIC of total WOMAC score.

	Odds ratio	95% Confidence interval
Age (y)	1.001	(0.9607-1.042)
Sex	0.8073	(0.3806-1.688)
BMI (kg/m^2^)	1.084	(0.9622-1.227)
Follow-up	0.9941	(0.9795-1.009)
Side	1.040	(0.4979-2.176)
Duration of symptoms	0.9824	(0.9482-1.019)
Prior procedures	1.275	(0.5929-2.681)
Presurgery HKA	0.8957	(0.6505-1.205)
Postsurgery HKA	1.266	(0.4840-3.209)
Tourniquet time	1.024	(0.9778-1.076)
Hospital stay	1.471	(0.7291-3.103)

### Revision, reoperation, and complications

Of the 246 patients available for follow-up, 4 were revised (Table 5). One for a periprosthetic joint infection, one for instability due to anterior cruciate ligament insufficiency, and 2 for progression of OA to the medial compartment. All were revised to a primary total knee arthroplasty. The average time to revision was 4.9 years (range: 2-8.5). The survivorship of the implant was 100% at 1-year (0/246), 99% at 2-years (1/246), and 98.4% at 5-years (3/187).

**Table 5:** Revisions, reoperations, and complications.

	(*n* = 255)
Follow-up (y)	6.6 ± 2.2 (range 2-10.8)
Lost to follow-up	4% (*n* = 9)
Implant revision rate	1.6% (*n* = 4)
PJI	1
Progression OA	2
Instability	1
Reoperation rate	2% (*n* = 5)
Partial meniscectomy	5
Complication rate	17.6% (*n* = 43)
Stiffness	38
Wound dehiscence	3
Neurapraxia	1
Intraoperative fracture of lateral femoral condyle	1

Five other reoperations were performed (2%), and those involved resection of a partial tear of the medial meniscus in all cases

Complications occurred in 17.6% (*n* = 43). This mainly involved postoperative stiffness of the knee (*n* = 38) requiring manipulation under anesthesia (MUA). Wound dehiscence occurred in 2 patients: one patient had a neurapraxia of the common peroneal nerve that fully recovered with conservative management, and one patient sustained an intraoperative fracture of the lateral femoral condyle that required open reduction and internal fixation.

## Discussion

We believe that this study reports the largest cohort of patients undergoing lateral UKA using the fixed-bearing Triathlon PKR implant and reports excellent survivorship (98.4% after 5 years) and good functional outcomes in 246 cases with a mean follow-up of 6.6 years. Age, BMI, and pre- and post-operative radiographic alignment were not found to correlate with the functional outcome. Our findings demonstrate that this implant offers an alternative option for the treatment of lateral unicompartmental OA with outcomes comparable to other fixed and mobile-bearing implants used for UKA of the lateral compartment.

Survivorship in lateral UKA has historically been inferior to medial UKA [4] and was attributed to early design difficulties arising from anatomical differences in relation to the lateral compartment [8]. But respondent shifts toward fixed-bearing and improved mobile-bearing implants have led to improved survivorship rates [13,14]. Survivorship for lateral UKA reported in large case series (>100 UKAs) ranges from 93 to 99% at 5 years and 85 to 91% at 10 years [7,14,[19], [20], [21]], with recent evidence also indicating that survivorship of fixed-bearing implants may be superior to mobile-bearing implants, even when specifically compared with the latest dome-shaped mobile-bearing design [13]. Despite these findings, there remains a paucity of data on fixed-bearing implants, mostly consisting of smaller (<50 UKAs) series [[22], [23], [24]]. The largest series of lateral UKAs (*n* = 268), including multiple fixed-bearing implant models (HLS Uni Evolution, Alpina Uni, ZUK, Sigma HP, and Uni Score), reported survivorship of 85% and 79% at 10 and 20 years, respectively [20]. Our cohort study is of a similar size and only includes a single fixed-bearing implant model, showing similar midterm implant survival rates for lateral UKA. New technology such as robotic-assisted and computer-navigated UKA may further improve survival rates due to more accurate component positioning [25].

This study specifically reports on the use of the Stryker Triathlon PKR fixed-bearing implant used specifically for lateral UKA. This implant was initially designed for medial UKA, which is not uncommon for lateral UKA implants [3]. For medial UKA, the Triathlon PKR implant has already been shown to demonstrate good clinical outcomes, with Middleton *et al* reporting a 9% revision rate after 5 years [26]. Only 2 implants had to be revised for aseptic loosening, which has been considered the main reason for the failure of fixed-bearing UKA implant designs [27]. We have not yet revised implants for aseptic loosening, but long-term follow-up remains essential to provide further insight into the occurrence of this potential mechanism of failure. Middleton *et al* revised most implants early (<2 years) and suggested that this might be due to a learning curve. For other medial UKA implants, a learning curve of 10-25 cases has been suggested [28]. The senior author, who uses the same implant for medial UKA, would suggest that a good baseline experience is obtained with the implant for medial UKA prior to commencing lateral UKA.

Patient selection for lateral UKA is also adapting. Historically, the Kozinn and Scott criteria were used for both medial and lateral UKA [29]. However, recent studies have shown that these criteria were overly restrictive considering advances in implant design and surgical technique [3,4]. Radiological asymptomatic patellofemoral joint OA was not found to negatively influence the outcome of lateral UKA [7,19,30,31], and recent studies also dispute previously considered contraindications, such as younger age and higher BMI [14,20,30]. The indication for a lateral UKA in our cohort was not restricted by these aforementioned factors, and using a multivariate regression model, we found no negative correlation between age or BMI and clinical outcome. Asymptomatic patellofemoral OA was not documented and therefore not included in the multivariate regression model.

The optimal postoperative alignment in lateral UKA remains subject to debate. Measuring alignment with HKA-axis on radiographs is 2-dimensional simplification of what in reality is a dynamic and 3-dimensional phenomenon. A consensus exists that overcorrection should be avoided due to the risk of progressive native compartment OA [6]. In medial UKA, a postoperative varus angle of 1-4° has been shown to be associated with improved functional outcome [32]. Due to anatomical and kinematic differences, this may not apply to the lateral compartment. Van der List *et al* demonstrated that valgus alignment of 3-7° correlated with significant functional improvement at 2-year follow-up in a small cohort of 39 lateral UKAs [33]. In our study, alignment was corrected to a mild valgus in 26% (*n* = 67), neutral in 35% (*n* = 88), and mild varus in 39% (*n* = 100), but we were unable to find a correlation between both pre- and post-operative alignment and clinical outcome. The correlation between alignment and revision was not studied due to the low number of revisions, but will remain of interest with long-term follow-up.

Further limitations of our study include the fact that this is a single-surgeon, single-institution cohort. UKAs make up a sizable proportion of the senior authors practice, comprising >60% of all knee arthroplasty cases, and this has been shown to improve clinical outcomes [34]. While the rate of MUA appears high, especially when compared to other series [8], this may reflect the opinion of the senior author, who has a low threshold for intervening in the early postoperative phase if he feels that the patient has not achieved maximum flexion and/or extension at 6-week follow-up. We are of the view that this significantly accelerates rehabilitation for patients following knee surgery, not only lateral UKA. Indeed, there may be clinical criteria that can aid in the decision-making process surrounding MUA.

Some have postulated that the kinematics of the lateral compartment, with posterior subluxation of femoral condyle in deep flexion contribute to the possible occurrence of stiffness, as there is a risk of overstuffing [35]. Overstuffing of the compartment and overcorrection of alignment should obviously be avoided, but in relation to postoperative stiffness in this series, it is our belief that this is not a causative factor. Lateral UKA, by its nature, is less likely to cause “mechanical” stiffness than medial UKA, due to increased sagittal motion during flexion in the lateral compartment, when the lateral collateral ligament allows up to 7 mm of distraction, compared to approximately 2 mm on the medial side [36]. In addition, analysis of the postoperative alignment in our series demonstrated no correction of the HKA-axis of >3° valgus, suggesting that overstuffing of the compartment was not an issue.

It is our contention that postoperative stiffness might be mainly a biological process, possibly inflammatory, due primarily to the formation of extra-articular adhesions, primarily in the peripatellar tissue, and not intra-articular factors, although it remains poorly understood. Also, the use of drains is controversial and has not been proven beneficial in total knee arthroplasty [37]. Finally, this is an independent cohort study, and some evidence has suggested that case series and cohort studies show better implant survival rates in comparison to joint registry data [14].

## Conclusions

This cohort study reports midterm clinical outcome and revision rates of the Stryker Triathlon PKR fixed-bearing implant for lateral UKA. A good clinical outcome with a low revision rate was found at midterm follow-up. Long-term clinical and radiological follow-up will remain essential to determine if the demonstrated clinical outcomes and low revision rates can be maintained, and may also provide further detail on the optimal postoperative alignment.

## Conflicts of interest

T. O'Donnell is a receiver of educational bursary (SISK Healthcare) for fellowship program and a paid consultant for Stryker (surgeon to surgeon) reference center; all other authors declare no potential conflicts of interest.

For full disclosure statements refer to https://doi.org/10.1016/j.artd.2023.101183.

## Appendix A Supplementary data

Conflict of Interest Statement for all the authors
